# Histo-epidemiological profile of breast cancers among women in the Central African Republic: about 174 cases

**DOI:** 10.1186/s12885-018-4256-2

**Published:** 2018-04-05

**Authors:** Augustin Balekouzou, Ping Yin, Cavin Epi Bekolo, Christian Maucler Pamatika, Marceline Djeintote, Sylvain Wilfrid Nambei, Bertrand Ba-Mpoutou, Dieubeni Rawago Mandjiza, Chang Shu, Minghui Yin, Tingting Qing, Boniface Koffi

**Affiliations:** 1 Department of Epidemiology and Biostatistics, School of Public Health, Tongji Medical College of Huazhong University of Sciences and Technology, Hangkong Road 13, Post Box 430030, Wuhan City, Hubei Province China; 2 Ministry of Public Health, Central Medical d’Arrondissement de Bare, Nkongsamba, Yaoundé Cameroon; 3 Hospital Laboratory Friendship Bangui, Independence Road, Bangui City, Central African Republic; 4 National Laboratory of Clinical Biology and Public Health, Abdel Nasser Road, Post Box 1426, Bangui City, Central African Republic; 5 Faculty of Health Sciences, University of Bangui, Martyr Road, Post Box 1383, Bangui City, Central African Republic

**Keywords:** Breast cancer, Women, Epidemiology, Histology, Central African Republic

## Abstract

**Background:**

Breast cancer (BC) is the most common cancer in women worldwide and leading cause of cancer deaths indeveloping countries. There is very limited data on BC in the Central African Republic. The purpose of this study was to describe the epidemiological and histopathological characteristics of BC in Bangui.

**Methods:**

This retrospective study reviewed cancer data registries and medical records from the Pathology Unit of the National Laboratory in Bangui and the General Surgery and Gyneacology service from 2003 to 2015. A questionnaire was designed to collect information and data was analysed using descriptive and inferential statistical methods.

**Results:**

In total, 174 cases of BC were recorded, with an average annual frequency of13.4 cases per year. The age of the women at diagnosis varied from 16 to 90 years with a median of 45.5 years and InterQuartile range (IQR) 18 years. The age group of 45–54 years represented the majority of the study population (*n* = 51, 29.3%).About 25.9%ofthe patients were non-educated and 85.6% lived in cities. Over 48 % of the women were housewives with a moderate economic status (*n* = 99, 56.9%). Sixty nine percent of the specimens received at the pathology unit were pieces of breast tumour. Invasive ductal carcinoma (*n* = 113, 64.9%) was the main histological form and most of the tumours were of Grade III (*n* = 14, 46.7%). The only imaging assessment was ultrasound performed in (*n* = 53, 30.4%) women. Surgery was performed in (*n* = 166, 95.4%) patients, while (*n* = 159, 91.4%) received complementary chemotherapy. At the end of the study, 84.5%of the cases had died, 12.1% were alive and 3.4% were considered “lost to follow-up”.

**Conclusion:**

BC is an important public health problem and affected most of the younger Central African women. Epidemiological and histological characteristics are more or less common to those described other developing countries. It is imperative to improve the awareness of health care institutions and women on the burden of BC, to carry out early screening of BC, and to strengthen the capacity of women’s health care system.

## Background

Breast cancer (BC) is the most commonly diagnosed cancer in women worldwide. It is the leading cause of death from cancer in most developing countries. With about1.7 million new cases diagnosed in 2012, it represents 23.0% of cancers in women and11.9% of all human cancers in the world [1]. The majority of newly diagnosed cases occurs in developing countries and affects more than one in ten women. Its incidence has increased significantly over the last two decades to 2.0% per year [2]. It is less ascertained in African and Asian women than in Europe and the United States [3]. BC is less common among the population under 25 years [4]. BC has a high morbidity and mortality when not diagnosed and treated early.

Incidence rates vary from one region to another throughout the world [5]. In Africa and East Asia it was 27 per 100,000, while in Europe it represented 96 per 100,000 [5]. In 2011, the annual incidence in Sub-Saharan Africa (SSA) was 22 per 100,000 women [6]. The incidence of BC increases from 35 years, peaking at 60 [7]. The study conducted in Mali in 2010, reported the regional variability with4.0% in Kenya, 10.0% in South African Republic and 16.0% in Senegal [8]. In Niger, BC represented 34.4% of all cancers in 2012, and was the first cancer in women [5]. In addition, Mali and Cameroon BC is ranked second among women after cervix cancer with 21.6% and 25.9% respectively [9].

According to the 2014 report by the World Health Organization (WHO), BC is ranked first of all cancers diagnosed in the CAR with 504 cases and the leading cause of death (24.5%) among Central African women [10, 11]. Nowadays, the survival rate has improved in developed countries through the practice of screening and progress observed in the areas of treatment. In most SSA countries, the fight against BC is still embryonic because there is no appropriate structure for the management of identified cases and human resources are insufficient. These reasons make it difficult to implement epidemiological surveillance of BC in this part of the globe.

Our multicenter study had as objective to identify all cases of BC diagnosed at the Pathology Unit of the National Laboratory in Bangui, to describe their epidemiological and histopathological characteristics in order to inform decisions aimed at better diagnosis and management of BC in CAR.

## Methods

The methodology used was the modified version of the Epidemiology of breast cancer: retrospective study in the Central African Republic already used by Augustin Balekouzou et al. [12].

### Study population

This was a retrospective study on the primary data collected from patients’ records at the Pathology Unit of the National Laboratory of Clinical Biology and Public Health in Bangui, the General Surgery and Gyneacology Services of the Friendship Hospital and the Community Hospital from September 2003 to September 2015. The cases were confirmed by histological or cytological analysis during the study period. The following participants were excluded: aged < 15 years old, not residing in the CAR territory, and all those whose diagnosis date was outside the study period. The study was approved by the Institutional Review Board of the School of Public Health, Tongji Medical College of Huazhong University of Science and Technology (IRB Approval File No.[2014] 09), and the Scientific Committee for study protocols validation of the Faculty of Health Sciences (FACSS) of the University of Bangui (No 2070 / UB / FACSS / CSCVPER / 16). Individual patient consent was not required due to the retrospective nature of this study.

### Data collection

A questionnaire was designed to collect information initially from the cancer register of the Pathology Unit of the National Laboratory; and from the medical records of patients in General Surgery and Gyneacology services. The information included: socio-demographic characteristics, such as age, occupation, economic status, education level, areas of residence, ethnic group, marital status, parity, menopausal and hormone therapy. The average annual frequency was calculated by the total number of BC diagnosed during the study period divided by the sum of the years during the same period. The clinical and histological characteristics were: location of the BC, nature of the sample, histological type of the tumour, grade of the tumour, treatment and course of the disease.

### Diagnosis of breast cancer

#### Clinical diagnosis

Clinical procedures for BC diagnosis were based on identifying a breast lump, skin texture, appearance or direction of the nipple, any unusual discharge and any rash or crusting of the nipple.

#### Pathology technique

The samples analyzed were mainly composed of chirurgical specimens (biopsy) and cytological specimens (aspiration by needles) fixed in 10% formalin and processed according to the usual techniques of paraffin embedding, microtome cutting and staining with hematoxylineosin. As for the detection of Ductal carcinoma in situ (DCIS), a breast ultrasound can show whether an abnormal area is solid (made of cells) or is a cyst filled with fluid. In the absence of imaging, a breast biopsy would be required to examine the tissue or cells under a microscope to search for cancer cells.

#### Classification criteria

Age was recoded as age groups (15 to 24 years, 25 to 34, 35 to 44, 45 to 54, 55 to 64, 65 to 74, 75 to 84, and > or = 85 years). Women who did not have a profession were classified as housewives.

The economic status was defined in terms of family income according to the international poverty threshold. Income is poor if it is below 2 dollars a day; moderate between 2 and 4 dollars; good between 5 and 10 dollars and excellent if it exceeds 10 dollars [13]. The residence was classified urban for those living in Bangui and rural for those living in the provinces (before diagnosis for BC). Ethnicity has been grouped into thirteen major groups in the Country (Banda, Gbaya, Mandjia, Sara, Yakoma, Gbanziri, Peulh, Goulha, Mbati, Ngbaka, Gbanou, Kaba and Zande). The level of education has been classified as illiterate, elementary, secondary and university. Marital status was classified as married, single, divorced and widowed. Parity was determined by the number of completed pregnancies in a woman surveyed before diagnosis. A woman who has never had a full-term pregnancy was considered nulliparous, while a woman with at least one to two full-term pregnancies was considered pauciparous.

The histopathogenic grade of Scarff-Bloom-Richardson (SBR) was used to specify the degree of cell differentiation of tumours on pathological examination [14].

#### Statistical analysis

Data analysis was performed using the Statistical Package for Social Sciences (SPSS Inc., Chicago, IL, USA) version20. Descriptive analysis was performed to characterise the demographic variables of the patients. InterQuartile range (IQR) and median were described for continuous variables with normal distribution and ranges for continuous variables with skewed distribution. Frequencies and proportions were used for categorical variables. Trend test was used to detect a significant change in trend with time.

## Results

### Socio-demographic characteristics of the sample

The socio-demographic characteristics of the patient were shown in Table 1. Globally, 174 cases of BC were identified during the study period. In total, 174 cases of BC were identified during the study period. The age at diagnosis for the cases ranged from 16 to 90 years with a median of 45.5 years and InterQuartile range (IQR) 18 years. Majority of the study population (*n* = 51, 29.3%) represented the age group of 45–54 years followed by 35–44 years (*n* = 49, 28.2%). Most patients (*n* = 85, 48.9%) were housewives with a moderate economic status (*n* = 99, 56.9%). Less than (*n* = 23, 13.2%) of the study population had a university degree and (*n* = 149, 85.6%) lived in cities. The most represented ethnic groups were the Banda (*n* = 32, 18.4%), followed by Mandja (*n* = 31, 17.8%) and Yakoma (*n* = 26, 14.9%).Unmarried women made up (*n* = 128/174, 73.6%) of the sample, however, a major proportion (*n* = 167, 96%) had given birth to at least one child. Menopausal women represented (*n* = 72/174, 44.1%) of the sample while small proportion (n = 14, 8.0%) had received hormonal therapy.

**Table 1:** Socio-demographic characteristics of the sample

Variables	Freq (174)	Percent (%)
Age-group (year)		
15–24	6	3.4
25–34	27	15.5
35–44	49	28.2
45–54	51	29.3
55–64	25	14.4
65–74	11	6.3
75–84	4	2.3
> =85	1	0.6
Mini, Median,	16; 45.5	
Maxi, IQR	90; 18	
Occupation		
Housewife	85	48.9
Employer	53	30.5
Shopping	30	17.2
Student	6	3.4
Economic Status		
Poor	24	13.8
Moderate	99	56.9
Good	48	27.6
Excellent	3	1.7
Education level		
None	45	25.9
Primary	57	32.8
Secondary	49	28.2
University	23	13.2
Residence		
Urban	149	85.6
Rural	25	14.4
Ethnic group		
Banda	32	18.4
Gbaya	22	12.6
Mandja	31	17.8
Sara	12	6.9
Yakoma	26	14.9
Gbanziri	10	5.7
Peulh	5	2.9
Goulha	0	0.0
Mbati	12	6.9
Ngbaka	8	4.6
Gbanou	2	1.1
Kaba	12	6.9
Zande	2	1.1
Marital status		
Married	42	24.1
Unmarried	128	73.6
Divorced	1	0.6
Widow	3	1.7
Parity		
< 1	7	4.0
> =1	167	96.0
Menopausal		
Yes	72	44.1
No	102	58.6
Hormone therapy		
Yes	14	8.0
No	160	92.0

*Freq* frequency, *Percent* percentage, *Mini* minimum, *Maxi* maximum, *IQR* InterQuartile

Employee includes all sectors: public and private. Poor family status (income < 2 dollars a day), moderate (income = 3 to 4 dollars a day), good (income = 5 to 10 dollars per day) and excellent (income> 10 dollars a day); Residence: Town (Bangui) and Rural (outside Bangui). Frequency was calculated by using Cross tabulation analyze

### Histological characteristics

**Fig. 1:**
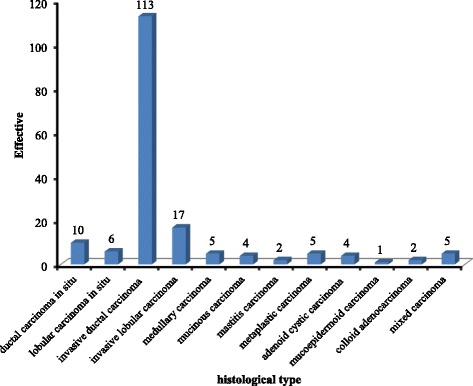
Distribution of tumours according to the histological type According to the Fig. 1, the predominant BC were invasive ductal carcinoma in (*n* = 113, 64.9%), invasive lobular carcinoma in (*n* = 17, 9.8%), ductal carcinoma in situ in (*n* = 10, 5.7%) and lobular carcinoma in situ in (*n* = 6, 3.4%). Medullary carcinoma and mixed carcinoma accounted for (*n* = 5, 2.8%) of each cases

According to the Table 2, the left breast was the most affected (80.0%) than the right breast (15.0%).The majority of the samples received and examined by the Pathology Unit were obtained in 69.0% of cases from breast lumpectomy and in 30.0% of cases from breast biopsies. The histological grade of ScarffBloom Richardson (SBR) was available for 30 patients (17.2%). SBR III was the main grade in the commonest histological types (46.7%). A total of (*n* = 166, 95.4%) of the patients underwent surgery, and (*n* = 159, 91.4%) underwent complementary chemotherapy. Breast ultrasonography was performed in (*n* = 53, 30.4%) women. The majority (*n* = 147, 84.5%) of the cases had died, (*n* = 21, 12.1%) remained alive and (*n* = 6, 3.4%) were considered “lost to follow up”. Sixty four point 9 % (*n* = 113, 64.9%) of all cases were invasive ductal carcinoma, followed by invasive lobular carcinoma (*n* = 17, 9.8%) and ductal carcinoma in situ (*n* = 10, 5.7%) according to the Fig. 1.

**Table 2:** Distribution of the location, nature of the sample, grade, treatment and outcome of the disease

Variables	Frequency	Percentage
Location		
Left breast	16	80.0
Right breast	3	15.0
Both breast	1	5.0
Total	20	100.0
Nature of the sample		
Mastectomy	2	1.0
Biopsy of the breast	52	30.0
Lumpectomy	120	69.0
Total	174	100.0
Scarff Bloom Richardson		
Grade I	6	20.0
Grade II	10	33.3
Grade III	14	46.7
Total	30	100.0
Treatment Chemotherapy		
Yes	159	91.4
No	15	8.6
Total	174	100.0
Surgery		
Yes	166	95.4
No	8	4.6
Total	174	100.0
Breast ultrasonography		
Yes	53	30.4
No	121	69.6
Total	174	100.0
Outcome		
Alive	21	12.1
Loss to follow up	6	3.4
Dead	147	84.5
Total	174	100.0

## Discussion

Access to histological diagnosis of BC is very problematic in most African countries like CAR. BC diagnosis is established after examination of the breast specimens for biopsy or cytology. In CAR, there is only one pathology unit located at Bangui the capital. This unit does not practice the essential immunohistochemistry to detect hormone receptors (ER, PR and HER2). In our study, the average annual frequency of BC reported during the period 2003 to 2015 was 13.38 cases per year. This proportion is slightly lower than that obtained by Darré et al.(2013) in Togo who found 22.5 cases per year [15]. The age of the women at diagnosis of BC varied from 16 to 90 years with a median of 45.5 years. Most of the patients (*n* = 100, 57.4%) were in the 35–44 and 50–54 age groups. Our results corroborate the studies carried out in Bangui by Koffi et al. (2004) and by Tong Li et al. (2016) in China, but slightly above the frequency reported by Marianne Dubard-Gault in 2014 [16–18]. However in Europe and the US, the average age of women with BC at the time of diagnosis was about 60 years, while in SSA countries was around 45 years. This difference could be explained first by a very short life expectancy than that of the population in industrialized countries, next by an information and awareness deficit on BC and finally by a lot of screening in women age 50 and over in the US and Europe [2]. These findings suggest that in Africa, BC affects women at an earlier age and at an advanced stage of diagnosis and has poor outcomes. Indeed, in the present study, about two-thirds of women with BC were in the younger age group and have presented with the disease at an advanced stage. The low frequency of BC in women in CAR may be due to under-medicalisation of the health care system, geographic and financial inaccessibility to health care facilities, diagnostic errors, cultural stress, disease stress, errors due to traditional medicine, ignorance of patients, shortage in cancer specialists and the absence of a program to control cancer in general and BC in particular. The same observation was reported in studies conducted by Koffi et al.(2003), Ben Gobrane et al., and Ekortard et al., in 2007 [19–21]. These factors constitute a barrier to the reduction of the burden of this disease in the CAR population. This is in contrast to data from developed countries where mammography screening and self-palpation is advocated. In addition, increased awareness of women has resulted in early detection of pathology. The medical-social support provided to women with BC ultimately resulted in increased adherence and survival rates for patients. This would also explain the under-representation of patients living in the rural area in this study population. It should also be pointed out that the high burden of BC in women aged 50 to 54 years old supports data from the literature which indicate that BC is a priori disease for older women.

In our study, we observed that malignant tumours predominated in the left breast. These results have also been reported by authors in previous studies in Africa [16, 22]. But different from findings obtained from other studies where their authors reported a predominance of cancer in the right breast [23, 24]. The frequency of the bilateral forms in our series is lower than that reported by Mengué S.(2002) in Gabon, and Khairy et al.(2005), in Saudi Arabia [23, 25]. Therefore, we do not have sufficient scientific evidence to justify the disparity of BC location in CAR women.

Sixty four point 9 % (*n* = 113, 64.9%) of all cases were invasive ductal carcinoma, followed by invasive lobular carcinoma (*n* = 17, 9.8%) and ductal carcinoma in situ (*n* = 10, 5.7%). The proportion of the more common histological forms (invasive ductal carcinoma) of BC that we recorded was slightly below that reported by Darré et al.(2013) in Togo, and Echimane et al.(2000) in Ivory Coast, who respectively reported 96.0% and 97.0% for cancers and 2.6% and 2.04% for sarcomas [15, 26]. In Cameroon (2013) and Ghana (2015), Engbang et al., and Edmund et al., found 74.3% and 91.6% of invasive ductal carcinomas followed by 4.3% and 3.1% of invasive lobular carcinoma [27, 28]. The same trends were observed in previous study carried out in Bangui by Koffi et al.,in 2004 [16]. However, the low DCIS rate could be justified due to the lack of mammography in CAR, the only tool for early detection of BC cases.

There was a high proportion of grade III in the order of 46.7%, followed by grade II (33.3%) and grade I (20.0%). In Ghana, more or less similar proportions have been reported by Ohene et al. (2012) [29]. These authors had found 53.7% of grade III tumours, 31.5% grade II tumours and 14.8% grade I tumours [30]. However, other studies have shown various proportions. A study by Essiben et al. (2013), found a trend in the following order of increasing frequency: grades II, I and III at the Gynecological Obstetric and Pediatric Hospital of Yaoundé [31]. In 2004, Koffi et al. in Bangui, noted that the grade II tumours were the most common with 58.4% of cases, while tumours grade I and II were in the same proportion (20.8%) [16]. These findings differ from those reported in Gabon by Meye et al.(2004) where Grade II tumours were less common than classes III and I [32].

According to several studies, the conservative treatment of BC is offered to some patients in SSA and only a few centres perform the sentinel node technique [33, 34]. The most common surgical treatment is mastectomy with systematic lymph node dissection because the majority of tumours are diagnosed in their T2-T4 stages with lymph node involvement. In addition, surgery must be compulsorily associated with other methods of treatment including chemotherapy, radiotherapy and hormone therapy; this for the control of the general cancerous disease [35]. In our patients, (*n* = 166/174, 95.4%) were treated with surgery and (*n* = 159/174, 91.4%) with complementary chemotherapy. Our results were in sharp contrast to studies conducted by Gakwaya et al. (2008), where only 26.0% of patients received chemotherapy and by Serdouma et al. (2006), where 66.3% had surgery [29, 36]. Given the socio-economic context in the CAR, chemotherapy, although indicated for the treatment of BC, is often faced by the problem of medicines for chemotherapy and the money to pay for them. Despite the large number of patients receiving chemotherapy, only 12.5%of the patients were able to complete their treatment cycles. Indeed, the cost of a cycle of chemotherapy was estimated at $ 260, resulting in the cost of a round of chemotherapy varying from $ 1040 to $ 1560. This cost was largely above the income of the patients according to the report of World Bank in 2015 which estimated the annual average income of the Central African is of the order of $ 338.7 [37]. This explains inaccessibility to chemotherapy especially for drugs not available in Bangui.

Certain limitations must be considered to explain the results of this study. First, in CAR there is no cancer registry and no national cancer control program. Therefore, the incidence of BC per year is difficult to determine and immuno-histochemical analysis of cases has not been performed. In terms of the representativeness of the data from this research, it can be pointed out that in CAR, there is only one pathology unit for the diagnosis of cancer and one cancer department for the medical management of cases. However, we believe that the results of this study can be reported to the general target population of CAR despite the low sampling. Secondly, depending on the type of this (retrospective) study, a significant amount of our data (*n* = 37, 14.0%) was removed due to lack of information as a result of poor records management. In addition, the death date of patients that should allow us to calculate the survival rate after diagnosis is not available. Finally, we believe that the results of this study will contribute to BC research in CAR.

## Conclusion

BC is an important public health problem and affected most of the younger Central African women. Epidemiological and histological characteristics are more or less common to those described other developing countries, with a younger age at onset, advanced stage at diagnosis, and/or poor prognosis. Most of the patients lived in an urban area. Invasive ductal carcinoma is a predominant form of BC diagnosed. Therefore, it is imperative to improve the awareness of health care institutions and women on the burden of BC, to carry out early screening of BC, and to strengthen the capacity of women’s health care system.

## Abbreviations

BC: Breast cancer
CAR: Central African Republic
SBR: Scarff Bloom Richardson
SPSS: Statistical Package for Social Sciences
SSA: Sub-Saharan African
WHO: World Health Organization
